# Necrotizing Fasciitis‐Induced Septic Shock due to *Aeromonas hydrophila* : A Case Report

**DOI:** 10.1002/ccr3.70150

**Published:** 2025-01-26

**Authors:** Gongjie Ye, Taohong Li, Yiru Weng, Zhouzhou Dong

**Affiliations:** ^1^ Department of Intensive Care Unit Lihuili Hospital affiliated to Ningbo University Ningbo Zhejiang Province China

**Keywords:** *Aeromonas hydrophila*, bacterial infection, necrotizing fasciitis, septic shock

## Abstract

*Aeromonas hydrophila* ( *A. hydrophila* ), a Gram‐negative bacterium commonly found in aquatic environments, has the capacity to be transmitted to humans through consumption of contaminated fish, water, or seafood. In this study, we present a case report concerning a 77‐year‐old female patient who experienced an acute exacerbation of chronic heart failure, subsequently developing severe septic shock due to necrotizing fasciitis caused by *A. hydrophila* . Infections caused by *A. hydrophila* are more prevalent during warmer months, particularly in regions characterized by dense aquaculture or the presence of natural water bodies. The excessive use of antibiotics in aquaculture has contributed to the emergence of antibiotic‐resistant *A. hydrophila* strains. The proliferation of multidrug‐resistant *A. hydrophila* presents a significant challenge for clinicians. In the context of atypical bacterial infections, the initial empiric therapy typically involves the use of third‐generation cephalosporins in conjunction with quinolone antibiotics. In the present case, the patient's successful recovery was achieved through the administration of a combination of cefoperazone/sulbactam and levofloxacin, as determined by sensitivity testing. This case study underscores the critical importance of considering *A. hydrophila* infection in patients with septic shock who present with necrotizing fasciitis.

**Summary**

- This case study present a 77‐year‐old patient with a medical history of hypertension and chronic cardiac insufficiency who developed severe septic shock and necrotizing fasciitis caused by *A. hydrophila* .
- The condition was effectively managed with a therapeutic regimen comprising cefoperazone/sulbactam and levofloxacin, following the identification of *A. hydrophila* in both blood and thigh puncture fluid samples through bacterial culture analysis.
-
*A. hydrophila* infections typically rise in warmer months, especially in areas with dense aquaculture or natural water bodies.
- Diagnosis required reviewing medical history and microbiological cultures, with timely antibiotic treatment improving results. The study emphasized considering *A. hydrophila* in necrotizing fasciitis cases.

## 1 Introduction

*A. hydrophila* , a Gram‐negative bacterium found in aquatic environments, exhibits pathogenicity towards amphibians, reptiles, and mammals. Transmission to humans can result from ingestion of contaminated food, trauma, or exposure to untreated water [1, 2]. The epidemiology of *A. hydrophila* is characterized by its widespread distribution in aquatic environments, particularly in warm‐water regions where aquaculture is prevalent. For instance, a study conducted in Punjab, Pakistan, found that *A. hydrophila* was present in 6.46% of *Channa marulius* and 6.25% of *Sperata sarwari* samples, with a high incidence of virulence genes detected in 95.7% and 94.4% of isolates, respectively [3]. *A. hydrophila* is an opportunistic pathogen [4]. In addition to its impact on fish, *A. hydrophila* poses zoonotic risks, as it can infect humans, particularly those with compromised immune systems. The zoonotic potential of *A. hydrophila* , a closely related species, has also been noted, suggesting that the epidemiological significance of Aeromonas species may be underestimated in both aquaculture and healthcare settings [5]. In the United States, *A. hydrophila* has been reported to cause approximately 10% of all cases of vibrio‐related infections, with a notable increase in cases during warmer months when water temperatures rise [6]. In tropical and subtropical regions, such as Southeast Asia, the prevalence of *A. hydrophila* infections can be significantly higher due to environmental factors that favor its growth, including warmer water temperatures and higher salinity levels. Moreover, a study conducted in India indicated that *A. hydrophila* was responsible for 15% of all cases of acute gastroenteritis in hospitalized patients, highlighting its role as a major pathogen in that region [7]. Consequently, *A. hydrophila* has emerged as a significant human pathogen. This report provides an examination of a case study involving a 77‐year‐old female patient who experienced an acute exacerbation of chronic heart failure, leading to the development of severe septic shock due to necrotizing fasciitis caused by *A. hydrophila* .

## 2 Case History and Clinical Findings

We report a 77‐year‐old female resident of Ningbo City in southeastern China, with a medical history of hypertension and chronic cardiac insufficiency, who was admitted to the hospital due to symptoms such as lower limb edema, decreased exercise tolerance, chest tightness, and persistent shortness of breath lasting for 24 h. The patient has a long‐standing history of severe lower limb edema and renal insufficiency for more than 20 years, with symptom control involving the use of diuretics and digoxin.

At the time of assessment, the patient's blood pressure was recorded at 142/68 mmHg, with a heart rate of 122 beats per min, a respiratory rate of 27 breaths per min, and a body temperature of 36.5°C. Laboratory evaluations revealed that routine hematological parameters and hepatic function tests were within normal reference ranges. However, the BNP (brain natriuretic peptide) level was markedly elevated at 3789.8 ng/mL, and the creatinine concentration was significantly increased at 485.9 μmol/L. A chest CT (computed tomography) scan demonstrated findings consistent with pulmonary edema. Collectively, these clinical and diagnostic findings are suggestive of acute heart failure and acute renal failure.

## 3 Methods: Investigations and Treatment

Due to the patient's severe medical condition, manifestations of renal failure and pulmonary edema were evident. Prompt initiation of continuous renal replacement therapy was undertaken to address volume overload. Subsequent to a two‐day regimen of renal replacement therapy, significant amelioration of the patient's heart failure symptoms was observed, characterized by increased urine output and normalization of renal function.

**FIGURE 1:**
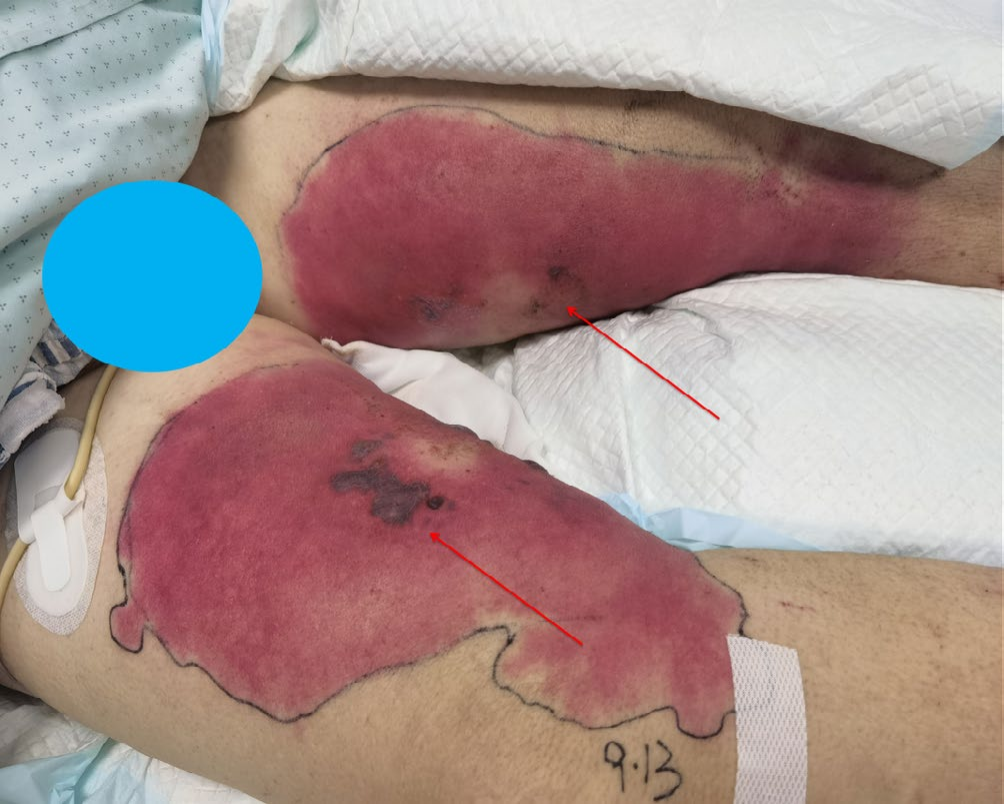
On the third day of hospitalization in the ICUs, the patient presented with black and purple blisters of varying sizes on the inner thigh, accompanied by a large red rash (as indicated by the arrow).

Nevertheless, complications arose during the patient's hospitalization. On the third day of admission to the ICU, the patient exhibited severe shock, drowsiness, and recurrent oliguria (urine output < 400 mL/24 h), necessitating the initiation of dialysis. At this time, the patient's SOFA (sequential organ failure assessment) score reached a value of 12. Additionally, elevated levels of CRP (C‐reactive protein) at 254.57 mg/L and PCT (procalcitonin) at 10.30 ng/mL were observed, indicating a potential pathogenic infection. Furthermore, the observation of black and purple blisters of varying sizes on the inner thighs, surrounded by large red rashes that did not blanch and were accompanied by an increase in skin temperature, was noted (Figure 1). Upon further analysis of the patient's medical records, it was discovered that two days before the onset of the illness, the patient engaged in a practice of cleaning the inner thighs with a board brush at a river, potentially leading to necrotizing fasciitis. Treatment was initiated with empirically selected antibiotics, specifically cefoperazone/sulbactam 2.0 g administered intravenously every 8 h in combination with linezolid 0.6 g administered intravenously every 8 h. Prior to the administration of antibiotics, blister puncture fluid and peripheral blood cultures were obtained for further examination.

**FIGURE 2:**
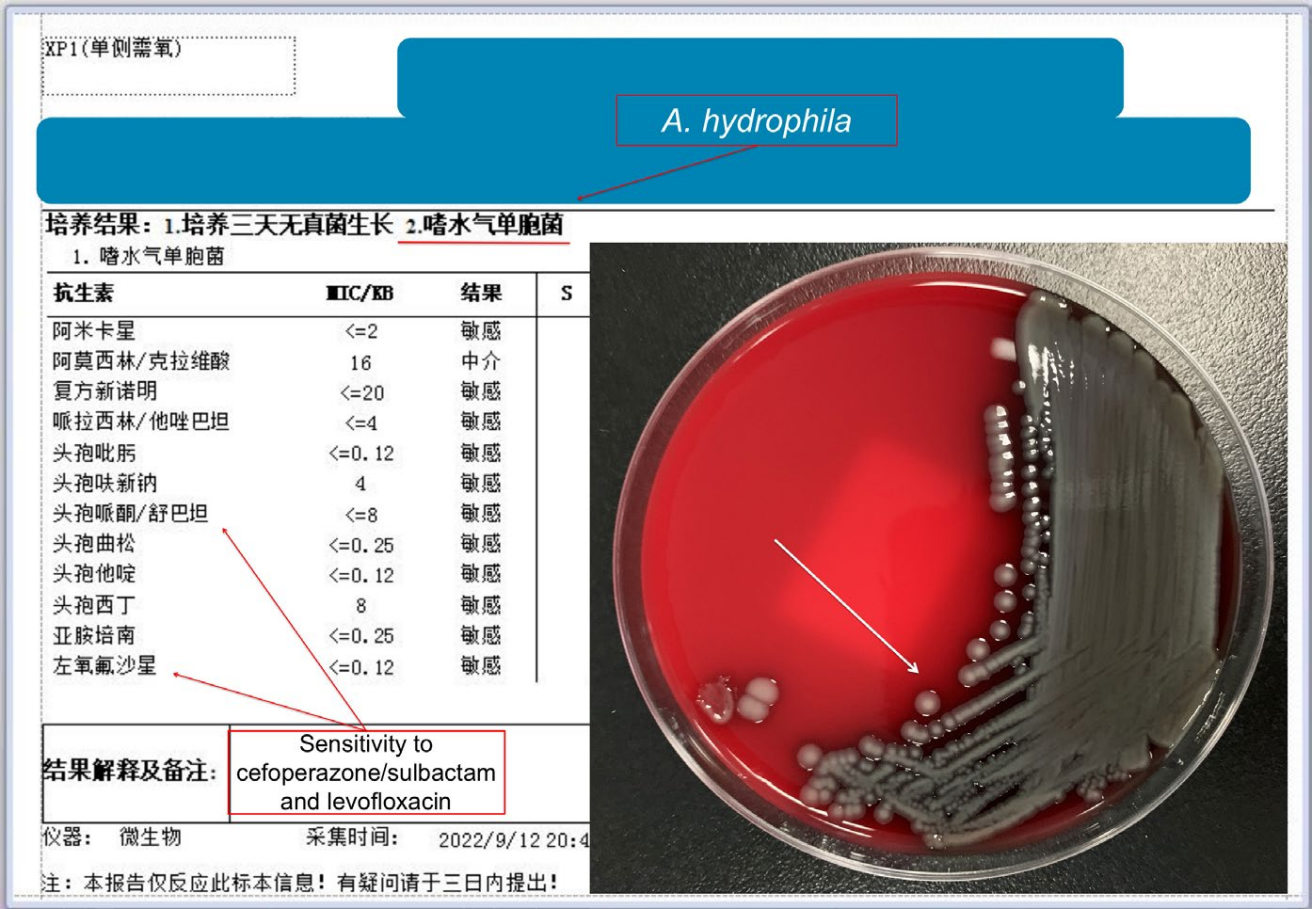
On the fifth day of admission to the ICUs, laboratory findings confirmed the presence of “ *A. hydrophila* ”, as evidenced by bacterial infiltration shadows indicated by white arrows. The sensitivity of the bacteria to cefoperazone/sulbactam and levofloxacin was highlighted by the red arrow.

However, despite the escalation of the patient's condition requiring high doses of vasoactive drugs to stabilize blood pressure, a positive development occurred on the fifth day of ICU admission when bacterial cultures identified the presence of “ *A. hydrophila* ” in both blood and thigh puncture fluid samples (Figure 2). The patient's diagnosis of septic shock was attributed to an infection with *A. hydrophila* . Antimicrobial susceptibility testing indicated that the bacterium exhibited a favorable response to cefoperazone/sulbactam and levofloxacin, leading the medical team to modify the anti‐infective treatment regimen accordingly.

## 4 Conclusion and Results

**FIGURE 3:**
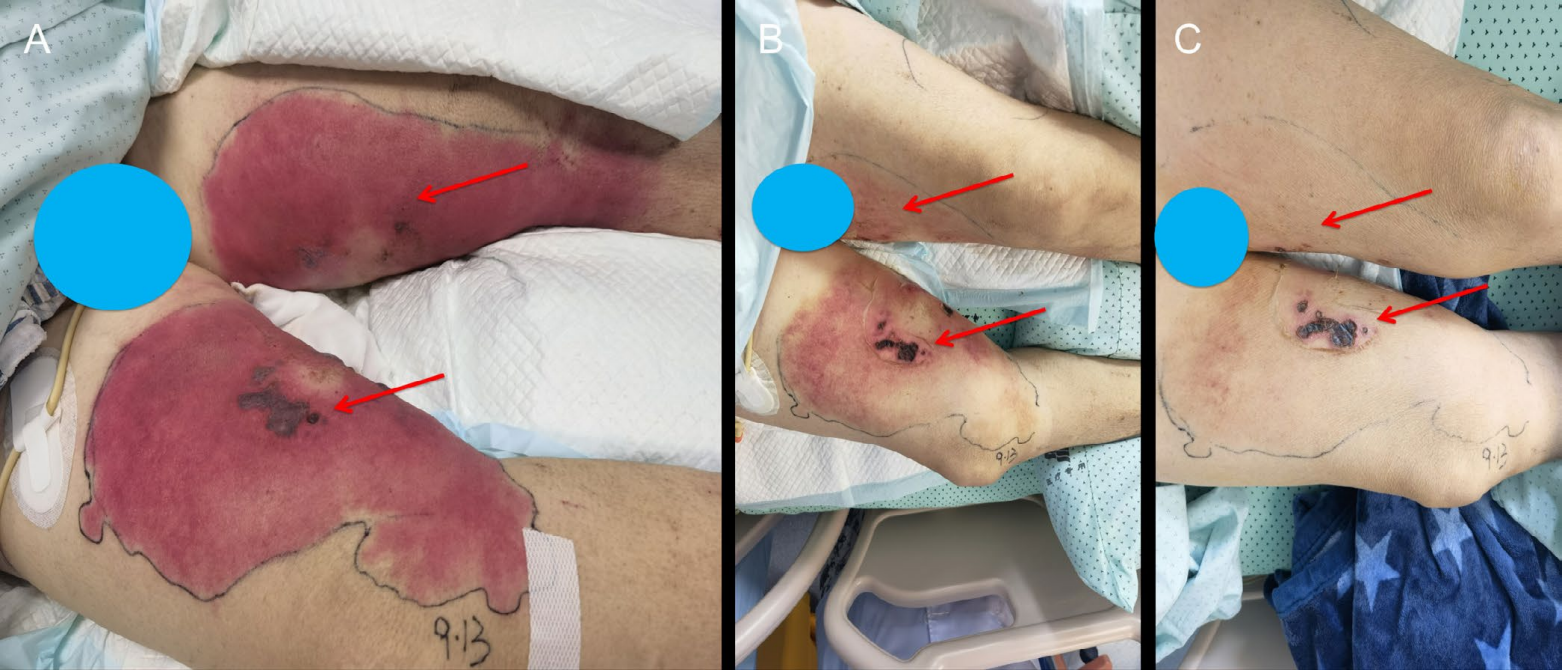
(A) On the third day of admission to the ICUs, the patient presented with black and purple blisters of varying sizes on the inner thigh, accompanied by a large red rash. (B) After 9 days of ICUs admission, the patient exhibited a reduction in blistering and the formation of scabs, along with resolution of the adjacent red rash. (C) Following a two‐week stay in the ICU, the patient's blisters progressed to scab formation and eventual healing, concomitant with the disappearance of the surrounding erythematous rash. The ecchymotic lesions present on the inner thighs of the patient resolved, while the surrounding erythematous rashes showed improvement.

Following the adjustment of the anti‐infection regimen to cefoperazone/sulbactam 2.0 g administered intravenously every 8 h in combination with levofloxacin 0.5 g administered intravenously every day, the patient's primary inflammatory markers exhibited significant improvement. Specifically, CRP levels decreased from 254.57 to 11.0 mg/L, reaching within the normal range, and PCT levels declined from 10.30 to 0.106 ng/mL, indicating a relatively low level. Additionally, the SOFA score was reduced from 12 to 2 points, and the patient's blood pressure gradually normalized. Furthermore, the ecchymotic lesions present on the inner thighs of the patient resolved, while the surrounding erythematous rashes showed improvement (Figure 3A–C). The administration of cefoperazone/sulbactam was carried out for a total of 14 days. Throughout the treatment period, we closely monitored the patient's response to the medication. Subsequently, the patient underwent successful tracheal extubation on the 11th day and was transferred to the general ward within the Infectious Diseases Department on the 14th day. Ultimately, the patient was discharged on the 20th day following admission to the intensive care unit. Post‐discharge, the patient has been regularly followed up at both the Infectious Disease and Cardiology clinics. Fortunately, there were no significant skin or cardiac complications observed. The patient showed a positive recovery trajectory.

## 5 Discussion

*A. hydrophila* , a Gram‐negative, rod‐shaped anaerobic bacterium, is a member of the Aeromonas genus known for its presence in fresh and brackish water, and in drinking water, wastewater, sewage, and food [8]. The first documented case of *A. hydrophila* as a human pathogen occurred in 1968 [9], and since then, the incidence of infections caused by pathogenic strains of this bacterium has been increasing. *A. hydrophila* has the potential to induce various infections including skin infections, pneumonia, gastrointestinal infections, pulmonary infections, hepatic infections, cardiac infections, dermatological infections, necrotizing fasciitis, and sepsis [10]. According to the data at hand, a notable percentage of skin infections and necrotizing fasciitis associated with *A. hydrophila* are linked to pre‐existing wounds or cuts, followed by exposure to water contaminated with the bacterium, leading to the emergence of serious dermatological ailments [11]. In the current context, the individual sustained a minor injury while cleaning their lower extremities, subsequently coming into contact with water contaminated with *A. hydrophila* , resulting in the development of necrotizing fasciitis and sepsis. In the clinical setting, the predominant skin and soft tissue infections observed are of bacterial etiology, encompassing conditions such as pustulosis, erysipelas, cellulitis, folliculitis, abscesses, and necrotizing fasciitis [12].

The infection caused by *A. hydrophila* primarily impacts immunocompromised individuals and some healthy individuals who have been exposed to contaminated water [13]. In individuals with compromised immune systems and comorbidities, infections caused by *A. hydrophila* are more likely to result in fatal necrotizing fasciitis and sepsis. Furthermore, the detection of *A. hydrophila* in the patient's blood culture indicates the onset of severe sepsis. The prevalence of *A. hydrophila* sepsis is relatively rare, yet its associated mortality rate ranges between 25% and 30%, often linked to compromised immune function and underlying medical conditions [14]. *A. hydrophila* has been linked to traumatic wound infections. A case involving an 89‐year‐old man who developed a wound infection after a car accident illustrates the pathogen's role in soft tissue infections. The infection was successfully treated with a combination of meropenem and levofloxacin, showcasing the importance of timely and appropriate antibiotic therapy in managing such infections [15]. Furthermore, a comparative study of clinical isolates revealed that *A. dhakensis*, often misidentified as *A. hydrophila* , is more virulent and prevalent in wound infections, indicating the need for accurate identification of Aeromonas species in clinical settings [16]. Given the patient's advanced age and medical history encompassing hypertension, atrial fibrillation, and chronic heart failure, there exists a heightened vulnerability to *A. hydrophila* invasion and subsequent sepsis development. Therefore, prompt diagnosis and initiation of antibiotic therapy are crucial in ensuring the patient's survival following the onset of sepsis. In this case, cefoperazone sulbactam and levofloxacin empiric treatments were found to be effective against *A. hydrophila* . The patient's condition improved significantly, leading to a successful recovery and subsequent discharge.

Necrotizing fasciitis is a severe soft tissue infection that can lead to septic shock, and *A. hydrophila* has emerged as a significant pathogen in this context [17]. Necrotizing fasciitis and skin infections induced by *A. hydrophila* are characterized by the presence of brown blisters, extensive rashes, and a propensity for evolving into erosive soft tissue infections [18]. *A. hydrophila* possesses several virulence factors contributing to its pathogenicity. One of the key factors is the type 6 secretion system (T6SS), which allows the bacterium to inject effector proteins directly into host cells, facilitating its survival and proliferation during infection. In particular, the TseC effector has been shown to play a crucial role in the virulence of *A. hydrophila* strains associated with necrotizing fasciitis [19]. In the case of *A. hydrophila* , sensitivity to both cephalosporins and quinolones allows for rapid relief of infection symptoms in patients [20]. However, it is important to recognize that the extensive use of antibiotics in freshwater fish farming has contributed to the development of multidrug‐resistant and potentially pan‐resistant strains of *A. hydrophila* [17]. *A. hydrophila* acquires and shares antibiotic resistance genes by transmitting mobile elements like plasmids, integrons, insertion sequences, and transposons [21].

The management of *A. hydrophila* infections requires a comprehensive approach, including accurate diagnosis, assessment of severity, and appropriate antibiotic therapy. In the case of multidrug‐resistant/pan‐resistant *A. hydrophila* , conducting antibiotic susceptibility testing in clinical settings is imperative for identifying effective antibiotics to combat the infection. Colistin, tetracyclines, and cefepime have shown potential efficacy against this resistant pathogen, and combination therapy with two different classes of antibiotics may also be considered [22]. In terms of established guidelines, the use of severity assessment scores, such as the SOFA score, can be instrumental in evaluating the severity of infections and guiding treatment decisions. The SOFA score helps clinicians assess the extent of organ dysfunction and can inform the need for empirical antibiotic therapy, particularly in severe cases of serious infections and severe community‐acquired pneumonia [23].

Empirical antibiotic regimens for *A. hydrophila* infections typically include broad‐spectrum antibiotics, as this organism is often resistant to multiple agents. The choice of antibiotics may be influenced by local resistance patterns and the patient's clinical status. Furthermore, the importance of antibiotic stewardship cannot be overstated. Overuse of broad‐spectrum antibiotics can lead to increased resistance, making it crucial to tailor therapy based on culture results and clinical response [24]. Furthermore, the contamination of water bodies with antibiotic residues from agricultural runoff and wastewater can facilitate the spread of resistant strains, thereby posing a dual threat to public health and environmental safety [25]. In light of these challenges, effective infection control measures are crucial. Public health initiatives must focus on monitoring and managing the quality of water in recreational and aquaculture settings to mitigate the risks associated with *A. hydrophila* . This includes implementing guidelines for water treatment and sanitation, and educating communities about the potential health risks linked to exposure to contaminated water [26].

## 6 Conclusion

This case study described a chronic heart failure patient who suffered severe septic shock and necrotizing fasciitis due to *A. hydrophila* . Successful recovery was achieved with targeted antibiotics (cefoperazone/sulbactam and levofloxacin). The rise of multidrug‐resistant *A. hydrophila* strains poses a major challenge for clinicians. Diagnosing *A. hydrophila* infection involves reviewing medical history and conducting microbiological cultures. Timely and suitable antibiotic treatment improves outcomes. This case study underscores the need to consider *A. hydrophila* in patients with necrotizing fasciitis and black blisters, especially if they have been exposed to contaminated water.

## Author Contributions

**Gongjie Ye:** writing – original draft. **Taohong Li:** investigation, writing – original draft. **Yiru Weng:** data curation. **Zhouzhou Dong:** writing – original draft, writing – review and editing.

## Ethics Statement

Written informed consent was obtained from the patient for the publication of this case report and accompanying images. A copy of the written consent is available for review by the Editor‐in‐Chief of this journal upon request.

## Consent

The authors have nothing to report.

## Conflicts of Interest

The authors declare no conflicts of interest.

## Data Availability

The data and figures that support the findings of this study are available from the corresponding author upon reasonable request.
